# Editorial: Alternative and complementary therapies to promote mental health and wellbeing for older adults

**DOI:** 10.3389/fpsyg.2023.1192239

**Published:** 2023-04-25

**Authors:** Meiling Qi, Cindy Jones

**Affiliations:** ^1^School of Nursing and Rehabilitation, Cheeloo College of Medicine, Shandong University, Shandong, China; ^2^Faculty of Health Sciences and Medicine, Bond University, Gold Coast, QLD, Australia; ^3^Menzies Health Institute Queensland, Griffith University, Brisbane, QLD, Australia

**Keywords:** alternative therapies, mental health, wellbeing, older adults, complementary approaches

Older adults can experience a reduction in their mental health and wellbeing due to aging, physical inactivity, and sedentary behavior. Poor mental health and reduced wellbeing could, in turn, have serious effects on older adults' emotional status and quality of life (Da Silva et al., 2022). Alternative and complementary approaches, which refer to non-mainstream medical practices such as traditional Chinese exercises (e.g., Baduanjin and Tai Chi), may help reduce mental issues and improve wellbeing in older adults (Groden et al., 2017; Marciniak et al., 2020; Qi et al., 2020; Jones et al., 2022). This Research Topic focused on (a) original articles that provide evidence and inform our understanding of the effects of alternative and complementary approaches, and (b) the various factors that influence the design of these strategies and therapies to improve the mental health and wellbeing of older adults.

Six original articles that incorporate various sample populations, including older adults with dementia, older migrants, and older employees were included in this Research Topic collection. To begin with, using a three groups pilot randomized controlled trial, Lin and Li examined the effects of a 12-week olfactory-based sensory stimulation program with cognitive training on the cognition and emotion of older adults with mild to moderate dementia with the board game and control groups. Olfactory stimulation training with cognitive training was found to significantly decrease plasma amyloid ß1-42 with the potential to improve cognitive function and decrease depression, suggesting that sensory stimulation based on olfactory stimuli can potentially be beneficial for older adults with dementia. Furthermore, other articles have highlighted factors such as family support and psychological and occupational aspects that should be considered while designing alternative and complementary interventions to enhance the mental health and wellbeing of older adults.

The internet is an extensive reservoir of resources that may considerably improve the daily lives of older adults. Dong, Meng, et al. performed a secondary analysis of the China General Social Survey (2010 to 2017) which highlighted the benefits that the Internet can have on the wellbeing of older adults. Internet use has significant positive impacts on physical and life satisfaction among older adults via increased social networking opportunities and health insurance participation. However, excessive and prolonged internet use also comes with new health challenges, including depression, anxiety, loneliness, and sleep disorders. Yang et al. found that internet addiction in older adults may be deterred through interactions with children to reduce their feelings of loneliness. When designing future alternative and complementary interventions that involve internet use, it may be beneficial to incorporate elements such as family interactions to prevent or alleviate internet addiction among older adults, thus enhancing their mental health and wellbeing.

Life satisfaction plays an important role in promoting the health condition of older adults. Hou et al. conducted regression analyses using bootstrapping methods and found that older migrants who suffered from higher levels of perceived stress and anxiety reported lower life satisfaction, whereas resilience could potentially counteract the negative effects of stress and anxiety on life satisfaction in this population. Incentives to promote the uptake and participation of social activities within the local community, such as participating in sports and engaging in physical exercise should be considered (Gao et al., 2014). To improve the health and wellbeing of older migrants, the emphasis on future alternative and complementary interventions may be placed on alleviating stress and anxiety, taking into consideration the unique challenges faced by older migrants. Another study conducted by Dong, Ling et al. explored the impact of caring for grandchildren on Chinese older adults' life satisfaction. Those who provide care for their grandchildren demonstrated higher life satisfaction due to increased perceived self-efficacy and diminished experiences of loneliness resulting from companionship provided by grandchildren. It is, therefore, important to consider these aspects in the design of alternative and complementary interventions aimed at enhancing the mental health and wellbeing of older adults.

With an aging population, there is an increased number of older adults who remain in the labor workforce. Successful aging at work refers to the ability of older employees to maintain health conditions, physical function, motivation, and work capacity. Zhao et al. examined work-family enrichment which is referred to as “*the positive aspect of the infiltration between work and family*.” It is suggested that the accumulation of resources in the workplace can result in a positive mood, leading to enhanced family harmony and life satisfaction, also known as work-to-family enrichment. Reversely, the accumulation of resources in the family domain can improve work quality, known as family-to-work enrichment. Both family-to-work and work-to-family enrichment were positively associated with successful aging at work in the Chinese context. Thus, both family and work-related factors should be considered when developing alternative and complementary interventions to improve the health and wellbeing of older employees at home and in the workplace.

In conclusion, this Research Topic collection sheds light on the potential of olfactory stimulation with cognitive training in improving the mental health and wellbeing of older adults. When designing alternative and complementary interventions for this demography and aspects for consideration, factors such as family support and psychological and occupational considerations should be taken into account. Additionally, as the working population continues to age, experience high levels of stress in the workplace, and becomes susceptible to physiological and psychological health issues, there is a need for further research to consider both work and family resources in developing effective strategies targeted at enhancing health and wellbeing of older employees.

## Author contributions

MQ was primarily responsible for the preparation of the draft and subsequent revision prior to publication. CJ critically reviewed, edited, and revised the editorial. All authors substantially contributed to the editorial and read and agreed to the submitted version of the editorial.
